# Accessible Neurobehavioral Anger-Related Markers for Vulnerability to Post-Traumatic Stress Symptoms in a Population of Male Soldiers

**DOI:** 10.3389/fnbeh.2017.00038

**Published:** 2017-03-07

**Authors:** Tamar Lin, Gadi Gilam, Gal Raz, Ayelet Or-Borichev, Yair Bar-Haim, Eyal Fruchter, Talma Hendler

**Affiliations:** ^1^The Tel-Aviv Center for Brain Functions, Wohl Institute for Advanced Imaging, Tel Aviv Sourasky Medical CenterTel Aviv, Israel; ^2^School of Psychological Sciences, Tel-Aviv UniversityTel Aviv, Israel; ^3^The Department of Film and Television, Tel-Aviv UniversityTel Aviv, Israel; ^4^Sagol School of Neuroscience, Tel Aviv UniversityTel Aviv, Israel; ^5^Division of Mental Health, Medical Corps, Israel Defense ForcesTel Hashomer, Israel; ^6^Department of Physiology and Pharmacology, Sackler Faculty of Medicine, Tel Aviv UniversityTel Aviv, Israel

**Keywords:** anger, stress symptoms, PTSD, amygdala, biomarker, EEG, fMRI, electrical fingerprint

## Abstract

Identifying vulnerable individuals prone to develop post-traumatic stress symptoms (PTSS) is of paramount importance, especially in populations at high risk for stress exposure such as combat soldiers. While several neural and psychological risk factors are known, no post-traumatic stress disorder (PTSD) biomarker has yet progressed to clinical use. Here we present novel and clinically applicable anger-related neurobehavioral risk markers for military-related PTSS in a large cohort of Israeli soldiers. The psychological, electrophysiological and neural (Simultaneous recording of scalp electroencephalography [EEG] and functional magnetic resonance imaging [fMRI]) reaction to an anger-inducing film were measured prior to advanced military training and PTSS were recorded at 1-year follow-up. Limbic modulation was measured using a novel approach that monitors amygdala modulation using fMRI-inspired EEG, hereafter termed amygdala electrical fingerprint (amyg-EFP). Inter-subject correlation (ISC) analysis on fMRI data indicated that during movie viewing participants’ brain activity was synchronized in limbic regions including the amygdala. Self-reported state-anger and amyg-EFP modulation successfully predicted PTSS levels. State-anger significantly accounted for 20% of the variance in PTSS, and amyg-EFP signal modulation significantly accounted for additional 15% of the variance. Our study was limited by the moderate PTSS levels and lack of systematic baseline symptoms assessment. These results suggest that pre-stress neurobehavioral measures of anger may predict risk for later PTSS, pointing to anger-related vulnerability factors that can be measured efficiently and at a low cost before stress exposure. Possible mechanisms underlying the association between the anger response and risk for PTSS are discussed.

## Introduction

Identifying vulnerable individuals prone to develop post-traumatic stress symptoms (PTSS) is of paramount importance, especially in populations at high risk for prolonged and repeated stress exposures such as combat soldiers. Accumulating evidence indicate that anger is an individual tendency associated with stress-related psychopathology including PTSS (Meffert et al., 2008; Lancaster et al., 2011; American Psychiatric Association, 2013). However, research on the predictive relation between anger and PTSS is scarce. Few studies show that levels of anger experienced at the time of trauma (i.e., state-anger) predict later posttraumatic stress (Feeny et al., 2000; Ehlers et al., 2003; Jayasinghe et al., 2008). However, these aforementioned studies suffer from two major limitations: (1) anger was measured using self-report questionnaires; and (2) anger was measured in the aftermath of trauma, plausibly reflecting an early trace for the emerging disorder rather than a predisposing risk factor. This calls for an independent measure of evoked anger experience (e.g., brain response) that does not rely on self-report and assessed prior to prolonged stress exposure.

Most neuroimaging studies in PTSS focus on the fear response (Jovanovic and Ressler, 2010), pointing to heightened amygdala response to threat as a predisposing factor (Admon et al., 2009, 2013; McLaughlin et al., 2014; Swartz et al., 2015). The amygdala indeed plays a crucial role in threat detection, fear learning and fear expression (Pitman et al., 2012), but it appears to be activated by a whole range of other emotions, including anger (Fulwiler et al., 2012; Adolphs, 2013; Gilam and Hendler, 2015). An underlying role of the amygdala in anger expression is suggested by this region’s general role in the detection of threat, which may be relevant to anger experience, as well as to fear (Berkowitz, 1990). Abnormal amygdala activity may be specifically involved in unregulated anger generation and aggression. Indeed, neuroimaging studies demonstrate altered amygdala activity and connectivity in individuals with aggressive tendencies (Dougherty et al., 2004; Coccaro et al., 2007; Beaver et al., 2008; Passamonti et al., 2008). However, in these studies, as in most neuroimaging studies, the response to anger was evoked by the presentation of emotional faces (for review, see Gilam and Hendler, 2015), preventing the full capture of the complex dynamic nature of the experience of anger, thus disregarding a key parameter that could be important to stress vulnerability.

Conversely, prolonged naturalistic evocation of anger, such as with film excerpts, consisting of dynamically changing emotional cues, may better capture context-related on-going affective processing (Gross and Levenson, 1995). This type of stimulation approach can reliably infer both the dynamics of evolved emotional experience (Nummenmaa et al., 2012; Raz et al., 2012) as well as their neural representation in the brain (Hasson et al., 2004; Jääskeläinen et al., 2008; Nummenmaa et al., 2012; Abrams et al., 2013; Raz and Hendler, 2014). Thus, the use of such a naturalistic dynamic stimuli facilitates ecologically relevant elicitation of robust emotions and the mapping of neural responses that are evoked during the unfolding of the emotional experience.

**Figure 1:**
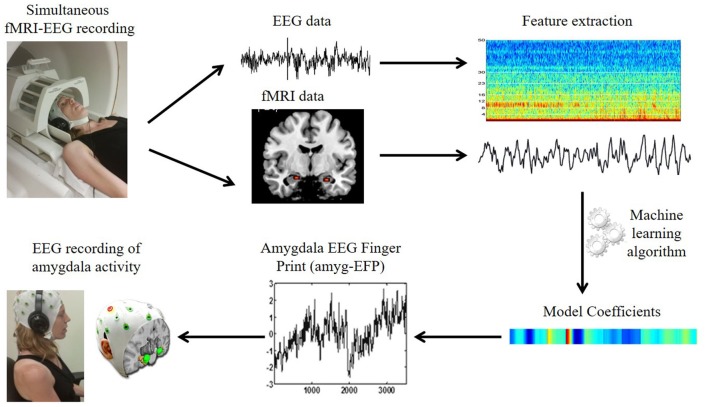
**The amygdala electrical fingerprint (amyg-EFP) prediction model.** Scheme illustrating EFP model construction. Electroencephalography (EEG) and functional magnetic resonance imaging (fMRI) data were acquired simultaneously. The fMRI time course of the amygdala and the time-frequency matrix obtained from the EEG data were used to calculate model coefficients using machine learning algorithms. This resulted in an EEG model predicting amygdala blood oxygenation level-dependent (BOLD) activity, which can be then used to record amygdala activity solely by EEG.

Despite the extensive search for PTSS biomarkers, there is a lack in clinically applicable risk markers (Schmidt et al., 2013, 2015). Such markers could be particularly valuable when high symptom load is evident following traumatic exposure and there is uncertainty with regard to full clinically significant post-traumatic stress disorder (PTSD). Continuous and regular monitoring of a predictive marker could improve early diagnosis and provide more effective treatment (Schmidt et al., 2013). One possible reason for the lack of such monitoring is that to date activity in deep subcortical regions, such as the amygdala, is typically measured by functional magnetic resonance imaging (fMRI); a method with high spatial resolution though expensive and inaccessible, thus restricting the translation of neuroimaging findings into clinical practice. Conversely, electroencephalography (EEG) is a relatively inexpensive mobile brain imaging technique, but inept from recording from deep limbic regions including the amygdala due to its poor spatial resolution (Nunez and Srinivasan, 2006). An approach that exploits the advantages of both neuroimaging methods has been recently developed in our lab (Meir-Hasson et al., 2014, 2016; Keynan et al., 2016). This approach relies on the application of advanced machine learning algorithms on EEG data acquired simultaneously with fMRI to produce an fMRI-inspired EEG model of amygdala activity; hereby termed *“amygdala-Electrical Finger Print”* (amyg-EFP; Figure 1). In a recent simultaneous EEG/fMRI experiment we validated the amyg-EFP as a reliable predictor of amygdala fMRI-blood oxygenation level-dependent (BOLD) activity, but also demonstrated that it may reflect the activity of a network of regions, including areas of the limbic and salience systems in which the amygdala is considered a major hub (Keynan et al., 2016). Importantly, we have shown that neuro-modulation guided by real-time monitoring of the amyg-EFP using neurofeedback correlated with deeply located limbic activity and has improved implicit emotion regulation among healthy individuals (Keynan et al., 2016), signifying its clinical applicability to traumatic stress.

The current study aims to identify novel and clinically applicable neurobehavioral anger-related markers for military-related PTSS in a large cohort of *a priori* healthy Israeli soldiers. For that, the use of the amyg-EFP signal to characterize the neural response to anger was examined. To capture the expected dynamic of the induced anger state, we used an anger provoking film excerpt taken from an Israeli documentary. State-anger and simultaneous EEG/fMRI during passive viewing of the film were measured prior to combat military training, and PTSS levels were obtained at 1-year follow-up after soldiers were exposed to chronic military stress. A common model of the amyg-EFP was then calculated following acquisition of individual EEG recordings. To promote the understanding of amygdala’s role in anger and PTSD, the association between amyg-EFP modulation during the film and selective attention to angry faces was measured by an established dot-probe task (Mogg and Bradley, 1999; Bar-Haim et al., 2007). We expected that the film would evoke anger experience that would be indicated in increased amygdala modulation both in fMRI-BOLD and in the common model of the amyg-EFP signal and would correspond to selective attention to angry facial expressions. We further predicted that the limbic hyperactivity, as measured by the amyg-EFP, would confer higher risk for PTSS beyond the predictive value of self-reported state-anger.

## Materials and Methods

### Participants

Participants were 50 male *a priori* healthy Israel Defense Forces (IDF) soldiers (mean age 18.86 ± 0.99) trained in a combat unit. This sample is similar to that reported in Gilam et al. (2017). Participants were recruited to the study during the first 2 weeks after military service enlistment. As part of the recruitment process, the soldiers were given a lecture at their military bases, explaining the goal of the study and its procedures. Soldiers who voluntarily agreed to participate in this study and met the inclusion/exclusion criteria were included in the study. PTSS were obtained a year later, following intensive and advanced combat training. During this training, participants were exposed to a wide variety of stressful physical and psychological demands, including sleep restrictions, prolonged periods of physical survival challenges, face-to-face combat training and counter-terrorism combat course, all of which have been shown to affect well-being (Gomez-Merino et al., 2005). Participants were asked to complete a questionnaire with several items regarding life history of traumatic and significant experiences and illnesses. These included specific questions regarding any severe mental and/or physical illnesses, and/or hospitalizations of the participant and his close family, as well as an open question in which participants were asked to describe any traumatic and/or significant experience that occurred throughout their civilian life prior to their military service. According to this, participants had no reported history of psychiatric or neurological disorders, no current use of psychoactive drugs, no family history of major psychiatric disorders, and no incidence of childhood abuse or potentially traumatic events before military enrolment. One soldier was discarded from the study since he decided to abort participation. This study was carried out in accordance with the recommendations of Declaration of Helsinki. All participants were recruited on a voluntary basis and provided written informed consent approved by Tel Aviv Sourasky Medical Center Ethics Committee and the IDF’s Ethics Board. The protocol was approved by the Tel Aviv Sourasky Medical Center Ethics Committee and the IDF’s Ethics Board.

### Anger Inducing Film Excerpt

A 5.21 min film excerpt taken from a documentary film *“Avenge but one of my two eyes”* (Mograbi, 2005) was used to induce anger (Raz et al., 2016). The clip introduces a political confrontation between the filmmaker and a group of soldiers who prevent the return home of a group of Palestinian children from school. The display of the film excerpt was preceded and followed by a 30-s epoch during which participants passively gazed at an all-black slide.

### Assessment of State-Anger

The intensity of anger as an emotional state was measured using the State Trait Anger Expression Inventory (STAXI-2; Spielberger et al., 1999). The questionnaire was administered following passive viewing of the anger inducing film excerpt.

### Retrospective Self-Reporting of Emotional Experience

#### Emotion Label Rating

An inventory containing 76 emotion labels was created on the basis of a comprehensive list of emotion words (Shaver et al., 1987). The labels were translated to Hebrew and presented along with their corresponding annotations, adapted from the Rav-Milim Hebrew dictionary (Choueka, 1997). After EEG-fMRI recording, participants rated the intensity to which they experienced each labeled emotion on a seven point Likert-like scale.

#### Continuous Emotion Rating

Following the EEG session, participants were presented with the same film excerpt and continuously rated the intensity of anger experienced while watching the clip during EEG recording, i.e., retrospectively. By using the computer-mouse, participants indicated changes in the intensity of anger felt in respect with a vertical scale continuously presented on the screen. The scale included seven levels of anger from neutral to very deep, each containing 3° of change (21° in total). The feedback was sampled at the rate of 10 Hz. This retrospective rating approach was successfully applied previously using cinematic material (Raz et al., 2012). A 100 s time window in which the averaged anger rating across all participants reached its maximum (second 189–288; hereby termed high-anger period) was considered as the most emotionally intensive period of the film. Similarly, a low-anger time window was established as the minimum averaged rating during the first 100 s (hereby termed low-anger period).

### Selective Attention to Angry Face

Attention bias to angry faces was evaluated with the dot-probe task (Mogg and Bradley, 1999) using E-Prime version 1.0 (Sharpsburg, PA, USA). In the dot-probe task, two face stimuli, one angry and one neutral, were shown briefly in each trial, and their offset was followed by a probe in the location just occupied by one of these faces. Stimuli were 128 angry/neutral face pairs and 32 neutral/neutral face pairs resented in a randomized order. Each trial began with a 500 ms central fixation cross. Two vertically aligned faces then appeared for 500 ms. These were replaced by a target probe that appeared in either of the two locations vacated by the faces. Probe type was either the letter E or F, determined randomly in each trial. Participants were required to identify which of the two probe types appeared by pressing the corresponding key as quickly as possible without compromising accuracy. The inter-trial interval was between 1300 and 1600 ms. Trials with incorrect response, trials in which the response time was two standard deviations below or above the participant’s mean for a particular condition, and trials in which response time was faster than 150 ms were excluded from the analysis. Attention bias was calculated as the difference between the average response time to targets in neutral face locations and those in angry face locations. A bias score in the positive range, which reflects faster mean reaction time to targets appearing at the location of the anger stimuli, was termed vigilance. A bias score in the negative range reflects the opposite pattern, which was termed threat avoidance.

Forty-one participants underwent this task, since the task was not available during data acquisition of the first eight soldiers enrolled to this study and two datasets were discarded due to data acquisition malfunction. Participants were allocated to groups according to their direction of attention-bias: avoidance (*n* = 20) and vigilance (*n* = 19). A similar classification has been recently used in healthy and clinical samples (Price et al., 2011; Waters et al., 2012; Lin et al., 2015).

### Assessment of PTSS

Symptoms were evaluated using the military version of the 17-items PTSD Checklist (PCL; Weathers et al., 1993), which is specifically designated to assess symptoms related to stressful military experiences. Thirty-three participants completed this questionnaire. In our sample, PTSS scores ranged from asymptomatic to moderate subthreshold levels (*M* = 27.6, SD = 9.4, range: 17–47).

### Electrophysiological Data Acquisition and Analysis

Electrocardiography (ECG) was recorded continuously during fMRI scanning via a BrainAmp ExG MRI-compatible system (BrainProducts, Munich, Germany). The sampling rate was 5000 Hz. For each participant, bipolar Ag/AgCl electrodes were attached to the right and left side of the chest. Preprocessing of the ECG signal and RR interval analysis was performed as described in Raz et al. (2012). Briefly, FASTR algorithm (Niazy et al., 2005) implemented in FMRIB plug-in for EEGLAB (Delorme and Makeig, 2004) was used for MR gradients artifacts removal. R peaks were detected using the FMRIB plug-in and manually corrected for mis-detection (average correction rate 0.8%). RR intervals were used to derive a beats-per minute heart rate (HR) index. HR time series were created as follows: the time points between two subsequent R components received a value, which corresponds to the duration of the previous RR interval. The data was then resampled with time bins of 1 s. Six datasets were discarded due to technical malfunctions, resulting in 43 datasets.

### fMRI Data Acquisition and Analysis

Brain imaging was performed by a GE 3T Signa Excite scanner using an 8-channel head coil at the Wohl Institute for Advanced Imaging, Tel-Aviv Sourasky Medical Center. Functional whole-brain scans were performed with gradient echo planar imaging (EPI) sequence of functional T2*-weighted images (TR/TE = 3000/35 ms; flip angle = 90°; FOV = 200 × 200 mm; slice thickness = 3 mm; no gap; 39 interleaved top-to-bottom axial slices per volume). Anatomical T1-weighted 3D axial spoiled gradient (SPGR) echo sequences (TR/TE = 7.92/2.98 ms; flip angle = 15°; FOV = 256 × 256 mm; slice thickness = 1 mm) were acquired to provide high-resolution structural images. Active noise canceling headphones (Optoacoustics, Moshav Mazor, Israel) were used. Preprocessing was performed using Brain Voyager QX version 2.1.4. Head motions were detected and corrected using trilinear and sinc interpolations, respectively, applying rigid body transformations with three translation and three rotation parameters. The data were smoothed temporally using a linear trend removal with a high pass filter of 0.008 Hz. Spatial smoothing with a 6 mm FWHM kernel was applied. To avoid the confounding effect of fluctuations in the whole-brain BOLD signal, for each TR, each voxel was scaled by the global mean at that time point. Anatomical SPGR data were standardized to 1 × 1 × 1 mm and transformed into Talairach space. SPGR images were then manually co-registered with the corresponding functional maps. Ten data sets were discarded from analyses: eight due to exaggerated head motions (deviations >1.5 mm and 1.5° from the reference point) and two due to technical malfunctions. Thus, fMRI analyses were performed on 39 data sets.

### Inter-Subject Correlation (ISC) Analysis

Inter-subject correlation (ISC; Hasson et al., 2004; Nummenmaa et al., 2012), was computed for the time course of the entire movie. For each voxel, a two-sided *t*-test was performed on Fisher-Z transformed Pearson’s coefficients of the correlation between the BOLD time-series of a single subject with the average time series of all other subjects (*p* < 0.0001, Bonferroni corrected).

### EEG Data Acquisition and Analysis

EEG was recorded simultaneously during fMRI acquisition using an MR-compatible system including a 32-channel BrainCap electrode cap with sintered Ag/AgCl ring electrodes (30 EEG channels, 1 ECG channel and 1 EOG channel; Falk Minow Services, Herrsching, Breitbrunn, Germany), and BrainAmp-MR EEG amplifier (Brain Products, Munich, Germany). Raw EEG was sampled at 5 kHz and recorded using Brain Vision Recorder software (Brain Products, Munich, Germany). EEG analyses were performed using EEGLAB 6.01 software package (Schwartz Center for Computational Neuroscience, University of California, San Diego, CA, USA), MATLAB software and FMRIB plug-in for EEGLAB. Pre-processing of the EEG data consisted of MR gradient artifacts removal using a FASTR algorithm (Niazy et al., 2005), cardio-ballistic artifacts removal (University of Oxford Centre for fMRI) and ICA-based artifact correction (Srivastava et al., 2005). Nine soldiers had technical problems with EEG acquisition and their datasets were discarded, resulting in 40 EEG datasets.

### EFP Analysis

The EFP approach was applied and validated on an ROI extracted from the right amygdala (Talairach [20, −5, −17], Gaussian sphere radius 6 mm; Meir-Hasson et al., 2014, 2016; Keynan et al., 2016) and right inferior frontal gyrus (IFG; 920 anatomical 1 mm^3^ voxels surrounding Talairach coordinate *x* = 42, *y* = 25, *z* = −1). The right amygdala was chosen based on previous findings indicating its reactivity to intense stress (Kinreich et al., 2011) and its coordinates were based on the findings of a comprehensive meta-analysis of 162 emotion studies (Kober et al., 2008), classifying the amygdala as a member of a limbic cluster. The common amyg-EFP model was developed by applying an iterative machine learning approach on the individual models previously constructed (*n* = 20), searching for a common multidimensional model that will best predict the common amygdala BOLD activity distribution of all subjects. Hierarchical clustering was applied in order to select the individual models of the strongest predictive power. A one class ridge regression was used in order to find weighted coefficients that will have the best predictive power across all participants. Finally, cross validation was applied to find the electrode with highest predictive power. The same analysis was applied to a time/frequency representation of the EEG data extracted from electrode F4, resulting in estimated model coefficients that rely on spectral-frontal features that correlate with the right IFG activity (IFG-EFP). Amyg-EFP and IFG-EFP signal amplitude was sampled at the rate of 0.03 Hz. For each participant, an average amyg-EFP and amyg-IFG signals were calculated for the low-anger and high-anger periods, reflecting limbic and prefrontal response as measured by the EEG during the slightest and most angering periods of the film excerpt. The IFG model was recently developed in our lab and was used in this study to test for the specificity of the amygdala as a predictor for PTSS. The IFG is of great relevance to this study since this region has been implicated in a variety of tasks requiring cognitive and inhibitory control including emotion regulation (Tabibnia et al., 2011), with a specific involvement in the regulation of anger experience (Buhle et al., 2014; Gilam and Hendler, 2015).

## Results

### Behavioral and Electrophysiological Results of the Anger Inducing Film

**Figure 2:**
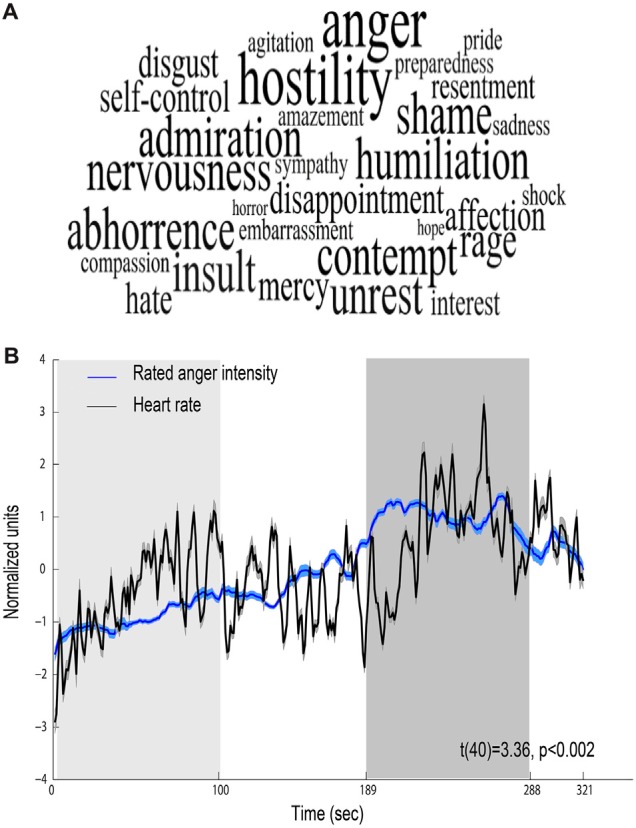
**Behavioral and physiological reactions to the film. (A)** Retrospective ratings—font size represents the median value of ratings higher than one (maximal size corresponds to a median score 5 out of 7). **(B)** Relations between behavioral and physiological indices. Mean scores of retrospective continuous rating of anger (blue, *N* = 39) and heart rate (HR; black, *N* = 43). Shaded regions around the waveform represent error bars. The 100 s of low-anger window with minimum anger rating across all participants is marked with a light gray rectangle and the high-anger time window is marked with a dark gray rectangle.

Behavioral ratings confirmed that the movie elicited anger, revealing that the most intensively experienced labels during the viewing of the film excerpt were anger and hostility (median score 5 out of 7; Figure 2A). Figure 2B shows mean time course obtained for all participants for the levels of anger intensity measured by retrospective continuous reports during a second viewing of the film. The figure also depicts continuous HR (beats per minute) response during the film, showing a physiological reaction to the film. Anger intensity and HR during the high-anger period were significantly higher than the intensity and HR measured at the low-anger period (anger intensity: *t*_(38)_ = 17.672, *p* < 0.001; HR: *t*_(42)_ = 3.9, *p* < 0.001), providing further support for an effective induction of anger measure both objectively and subjectively. Finally, for each participant we calculated Spearman correlation between the time courses of retrospective anger ratings and continuous HR time-series. A one sample *t*-test on individual Spearman correlation coefficients between these indices revealed that retrospective anger rating was positively correlated with the online HR modulation (Mean Spearman *r* = 0.12, *t*_(40)_ = 3.36, *p* < 0.002, *N* = 41; Figure 2B).

### Neural Results of the Anger Inducing Film

**Figure 3:**
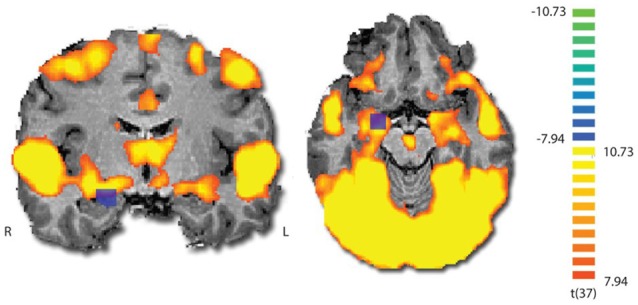
**Inter-subject correlation (ISC) map during film viewing (*n* = 39).** The map is thresholded at *p* < 0.0001 (Bonferroni corrected). The blue square indicates the region from which the BOLD signal was originally extracted to generate the common model of the amygdala EEG fingerprint.

To validate that the film excerpt effectively modulates amygdala activation, we used ISC analysis on the fMRI data, examining if amygdala responses become synchronized across participants. During movie viewing, the brain activity was highly time-locked across subjects in several brain regions (Figure 3). Largest ISCs were observed in the occipito-parietal visual cortices. However, statistically significant ISCs were also observed in limbic regions implicated in affective processing, including the amygdala and insula. Notably, there was a partial overlap between the region of the amygdala revealed in the ISC analysis and the area which was originally used (on a different group) to develop the amyg-EFP model (Meir-Hasson et al., 2014). To measure anger-related amygdala modulation using EEG, we calculated the time course of the amyg-EFP signal for each subject and associated with behavioral and clinical measures.

### Relation between Levels of Anger Intensity and Amyg-EFP Modulation

The behavioral and fMRI results indicate that the film excerpt induced an anger experience and activated the amygdala. To examine whether the anger intensity experienced during film viewing corresponds with limbic modulation, we calculated Spearman correlation between the time courses of anger ratings and amyg-EFP for each participant. A one sample *t*-test on individual Spearman correlation coefficients between the behavioral and amyg-EFP indices revealed a significant association (Mean Spearman *r* = 0.08, *t*_(38)_ = 2.54, *p* < 0.02, *N* = 39) further supporting the involvement of the amygdala in the anger response induced by the film excerpt.

### Relation between Selective Attention to Angry Faces and Amyg-EFP Modulation

**Figure 4:**
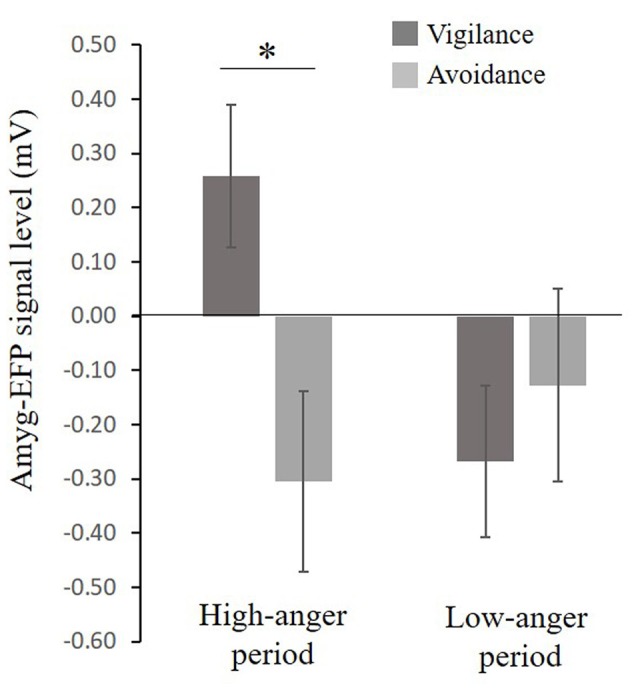
**Relationship between selective attention to angry faces and the EFP signal during the film.** A marginal significant interaction (*F*_(1,30)_ = 3.47, *p* = 0.07), showing that in the high-anger period, vigilant individuals had increased EFP signal (*N* = 17) compared to avoidance individuals (*N* = 15) *F*_(1,30)_ = 7.54, *p* < 0.02, marked by *. Bias groups did not differ in their EFP signal during the low-anger period *F*_(1,30)_ = 0.53, *p* = 0.46.

A considerable body of research suggests that anxiety might be associated with a pattern of selective attention toward threat (i.e., vigilance, see Bar-Haim et al., 2007) and with an unbalanced interplay between limbic and prefrontal cortex (PFC) function (Bishop, 2007, 2008). In an attempt to understand the role of the limbic system including the amygdala in anger and PTSS, participants completed the dot-probe task composed of angry (threat) and neutral faces outside the scanner. Participants were first stratified based on their attentional bias tendency to vigilance and avoidance groups. For the vigilance group (*N* = 19), mean attention bias was 18.7 ± 2.3 ms, a score significantly different from zero *t*_(18)_ = 8.1, *p* < 0.001, Cohen’s *d* = 1.86 and for the avoidance group (*N* = 20) mean attention bias was −15.1 ± 3.1 ms, a score significantly different from zero *t*_(14)_ = −4.8, *p* < 0.001, Cohen’s *d* = 1.23. The bias groups did not differ in PTSS *t*_(24)_ = −0.99, *p* = 0.33, Cohen’s *d* = 0.39 (Vigilance: *M* = 26.71, SE = 3.7, *N* = 14; Avoidance: *M* = 23.58, SE = 3.4, *N* = 12). To examine the relation between selective attention to angry faces and the amyg-EFP signal during the naturalistic angering film, a repeated-measures analysis of variance (ANOVA) was conducted with bias group (avoidance, vigilance) as a between-subject factor and period in the film (low-anger, high-anger) as a within-subject factor. No significant main effects of bias group *F*_(1,30)_ = 2.47, *p* = 0.12, $\eta_{\text{p}}^{2}$ = 0.07 and time *F*_(1,30)_ = 1.25, *p* = 0.27, $\eta_{\text{p}}^{2}$ = 0.04 were found. However, the interaction between these two variables was marginally significant *F*_(1,30)_ = 3.47, *p* = 0.07, $\eta_{\text{p}}^{2}$ = 0.10. Exploratory *post hoc* analyses revealed that in the high-anger period vigilant individuals had increased amyg-EFP signal (*M* = 0.25, SE = 0.13, *N* = 17) compared to avoidance individuals (*M* = −0.26, SE = 0.13, *N* = 15) *F*_(1,30)_ = 7.54, *p* < 0.02. There was no significant difference between bias groups during the low-anger period *F*_(1,30)_ = 0.53, *p* = 0.46 (Figure 4).

### The Predictive Contribution of Psychological and Biological Indicators of the Anger Response to PTSS Vulnerability

To assess whether pre-stress behavioral, neural and electrophysiological indices of anger predict PTSS following exposure, a hierarchical multiple regression analysis was applied. State-anger was entered to the model at the first step and amyg-EFP during the high-anger period was entered at the second step. State-anger before stress exposure entered in step 1 significantly accounted for 20% of the variance in PTSS at follow-up. Step 2 of the regression revealed that amyg-EFP signal before stress exposure accounted for additional 15% of the variance above and beyond the variance accounted by state-anger entered in step 1. The estimated coefficients for the regression model predicting the effects of the anger response before stress exposure on PTSS at follow-up are shown in Table 1A.

**Table 1:** **Regression predictors on post-traumatic stress symptoms (PTSS) following chronic military stress**.

Model (step)	Factors	Beta	*p*-value	*R*^2^	Tolerance
**A**
1	State-anger	0.454	0.010	0.206	
2	State-anger	0.793	0.001	0.354	0.561
	Amyg-EFP (high-anger period)	0.513	0.017		0.561
**B**
1	State-anger	0.454	0.010	0.206	
2	State-anger	0.610	0.007	0.245	0.615
	Amyg-EFP (low-anger period)	−0.252	0.238		0.615
**C**
1	State-anger	0.454	0.010	0.206	
2	State-anger	0.542	0.008	0.231	0.770
	IFG-EFP (high-anger period)	−0.182	0.344		0.770

*Hierarchical multiple regression analyses applied to assess prediction of PTSS following combat-training exposure. In three different models, state-anger was entered at the first step that significantly accounted for 20% of the variance in PTSS, and at the second step: **(A)** Amygdala electrical fingerprint (amyg-EFP) during the high-anger period; **(B)** amyg-EFP during the low-anger period; and **(C)** Inferior frontal gyrus (IFG)-EFP during the high-anger period; were added, respectively. Only amyg-EFP during the high-anger period significantly accounted for additional 15% of the variance in PTSS, above and beyond the variance accounted by state-anger. This emphasizes the specificity of the anger and amygdala reactivity during the viewing of an angering film in regards to the prediction of PTSS on year later following combat-training exposure*.

To confirm that the predictive value of amyg-EFP signal reflects the anger response, we performed the same analysis with amyg-EFP sampled during the low-anger period entered at the second step. The unique contribution of this variable was not significant and the addition of it to the model did not significantly increase the variance in PTSS explained by this regression model (Table 1B). Furthermore, to test whether the neural response is specific to the amygdala reflecting anger reactivity, an alternative model was tested with a different newly developed fMRI inspired EEG model that reflects the activity of the IFG, a region involved in anger regulation (Buhle et al., 2014; Gilam and Hendler, 2015). Similarly, the unique contribution of this variable was not significant and the addition of it to the model did not significantly increase the variance in PTSS explained by this regression model (Table 1C).

To further signify the necessity of accounting for the neural response, we performed additional regression analyses to test the predictive value of pre-stress anger rating and HR. The unique contribution of the subjective (anger rating) and objective (HR) anger reaction during the high-anger period was not significant either when adding it to state-anger in a second step or to amyg-EFP did not significantly increase the explained variance in PTSS (Table 2).

**Table 2:** **Regression models on PTSS testing the predictive value of the behavioral and physiological reaction to the film**.

Model (step)	Factors	Beta	*p*-value	*R*^2^	Tolerance
**A**
1	State-anger	0.454	0.010	0.206	
2	State-anger	0.467	0.009	0.223	0.990
	Anger rating (high-anger period)	−0.133	0.435		0.990
**B**
1	State-anger	0.454	0.010	0.206	
2	State-anger	0.471	0.009	0.230	0.990
	Heart rate (high-anger period)	0.146	0.388		0.990
**C**
1	State-anger	0.793	0.001	0.354	0.561
	Amyg-EFP (high-anger period)	0.513	0.017		0.561
2	State-anger	0.789	0.001	0.355	0.557
	Amyg-EFP (high-anger period)	0.500	0.026		0.527
	Anger rating (high-anger period)	−0.039	0.807		0.929
**D**
1	State-anger	0.793	0.001	0.354	0.561
	Amyg-EFP (high-anger period)	0.513	0.017		0.561
2	State-anger	0.833	0.001	0.391	0.550
	Amyg-EFP (high-anger period)	0.534	0.015		0.975
	Heart rate (high-anger period)	0.197	0.215		0.557

*Hierarchical multiple regression analyses applied to assess prediction of PTSS following combat-training exposure. In the first two models, state-anger was entered at the first step that significantly accounted for 20% of the variance in PTSS, and at the second step: **(A)** Anger rating of the film during the high-anger period; and **(B)** Heart-rate (HR) during the high-anger period; were added, respectively. No additional variance was accounted for above and beyond the variance accounted by state-anger. In the subsequent two models, state-anger and amyg-EFP during the high-anger period were entered at the first step, significantly accounting for 35% of the variance in PTSS, and at the second step: **(C)** Anger rating of the film during the high-anger period; and **(D)** HR during the high-anger period; were added, respectively. Again, no additional variance was accounted for above and beyond the variance accounted by state-anger and amyg-EFP during the high-anger period*.

## Discussion

The current study prospectively examines the predictive power of measurable psychological (state-anger) and neuronal (limbic response) indicators of the anger reaction in relation to PTSS in a unique military population at high risk for chronic stress exposure. The results indicate that both variables are significant predictors of subsequent PTSS. Specifically, individuals with elevated anger response (e.g., state-anger and amyg-EFP signal during a lab-generated angering experience) are at higher risk to develop symptoms following military stress.

The uniqueness of the study is underscored by the context in which amygdala activity was measured (anger rather than the common fear response), in the way anger was induced (dynamic naturalistic film excerpt rather than static angry faces) and by the neuroimaging method used to monitor limbic neural activity (EEG rather than fMRI). Our behavioral and physiological results indicate that the film used in this study effectively induced a dynamic anger response that propagated as the plot evolved and that this response was associated with the EEG limbic representation. ISC analysis demonstrated that during film viewing, participants’ brain activity was synchronized in sensory areas and in limbic circuits, confirming that the film evoked amygdala activation. These findings are compatible with earlier studies in clinically aggressive populations showing a link between anger and amygdala activity (Dougherty et al., 2004; Coccaro et al., 2007) and provide support for the use of the amyg-EFP signal to characterize the anger response.

The finding that the IFG-EFP signal did not predict PTSS suggests the specificity of the amygdala and indicated that a region related to anger reactivity, but not to anger control, is associated with stress symptoms following chronic stress. This corresponds with converging findings from imaging studies of the fear response, indicating that predisposing vulnerability is derived from regions related to emotion reactivity, while abnormal response of regions involved in emotion regulation are acquired factors that may lead to PTSS susceptibility (Admon et al., 2013). Our findings further show that the amyg-EFP predicted PTSS above and beyond the variability predicted by self-reported state-anger or by the physiological response measured by HR, signifying the importance of combining our neural measure as an accessible low cost marking for chronic stress vulnerability.

Converging evidence indicates that aberrant amygdala functioning is etiologically involved in PTSD development (Admon et al., 2009, 2013; McLaughlin et al., 2014; Swartz et al., 2015). However, currently, non-invasive imaging methods (e.g., EEG) are either limited by poor localization of deep brain areas such as the amygdala or by low accessibility and high costs (e.g., fMRI), questioning the translational capability of amygdala activity measurement to clinical practice. The current work demonstrates a novel accessible method to measure limbic modulation using EEG only that was correlated with provoked anger during the movie and successfully predicted increased risk for PTSS, providing an objective anger related risk marker that can be easily monitored. Notably, additional regression analyses demonstrated that the predictive value of amygdala response is specific to this region and restricted to a period when anger is experienced, increasing the validity of the methods used to induce anger and measure amygdala modulation. A recent neural model of PTSD asserts that heightened amygdala response mediates an exaggerated fear response that contributes to the symptom cluster of hyperarousal in PTSD (Admon et al., 2013; Norrholm et al., 2015). Along this line, amygdala hyperactivity may also mediate elevated aggressive impulse response under provoking environments (Coccaro et al., 2007), which may contribute to the PTSD symptom clusters of avoidance and emotional numbing (Foa et al., 1995). With this in mind, the well-established role of fear-related amygdala activity in PTSD (Pitman et al., 2012) should not be disregarded, warranting further investigation whether fear-related amyg-EFP signal can also be used as an effective biomarker for PTSS vulnerability.

The PTSS relevance of anger is indicated by cognitive models of PTSD postulating that anger could perpetuate the clinical status by contributing to perceptions of external threats (Ehlers and Clark, 2000), and by behavioral studies showing that trait and state-anger predict elevated PTSD symptoms following different types of stress exposure (Feeny et al., 2000; Ehlers et al., 2003; Jayasinghe et al., 2008; Meffert et al., 2008; Lommen et al., 2014). However, in these studies anger was only assessed using self-reported questionnaires. This study extends these findings by providing an objective neural marker that serves as a unique risk-index for PTSS above and beyond subjective self-report factors.

An ongoing challenge is characterizing the functional role of anger in PTSD, which may also explain the relationship between pre-stress anger and later risk for PTSS severity. According to the survival mode theory (Novaco and Chemtob, 2002), upon a threatening event, people tend to increase vigilance which entails a sense of loss of self-regulation and prompts anger that promotes aggression. While this process is beneficial in combat, in normative environments it becomes maladaptive. PTSD seems to cause greater readiness to perceive threat (Naim et al., 2014) and may thus enhance “survival mode” responding, thereby leading to excessive anger and dysregulation. The fear avoidance theory, on the other hand, postulates that anger is an emotional avoidance strategy comparable to cognitive avoidance strategies such as distraction (Foa et al., 1995), which are associated with high levels of PTSD symptoms (Dunmore et al., 2001). Therefore, being angry serves as a strategy to counteract the effects of fear and avoid the anxiety associated with PTSD and thus is enhanced in PTSD patients. An additional alternative suggests that anger may also potentiate threatening interpretations to ambiguous situations (Wenzel and Lystad, 2005; Barazzone and Davey, 2009), which may lead to negative appraisals of stressful situations that contribute to the persistence of PTSS (Ehlers and Clark, 2000).

Our finding that individuals with selective attention bias toward angry faces are characterized by an elevated amyg-EFP response during angering periods induced by the film seems to correspond with the survival mode theory; vigilant individuals are prompt to perceive threat which is associated with an elevated limbic response to an anger provoking environment. Further support for the relation between vigilance and anger is gained from a recent study showing the high locus coeruleus (LC) activity to interpersonal anger induced by a modified ultimatum game was associated with less anger regulation (Gilam et al., 2015). In fact, it has been recently shown that an increase in this LC activation during interpersonal anger following chronic stress was associated with the manifestation of PTSS (Gilam et al., 2017). Moreover, PTSD patients have increased LC activation during direct vs. averted eye-to-eye contact, which may be perceived as threat (Steuwe et al., 2014). Importantly, the amygdala and the LC have reciprocal anatomical connections (Samuels and Szabadi, 2008) and have been suggested to mediate threat detection and negative affect during the processing of anger (Gilam and Hendler, 2015). Furthermore, the current findings of an association between amygdala reactivity to anger and PTSS supports and broadens a previously suggest model on the development and manifestation of PTSD, in which hyper functionality of the amygdala in non-anger contexts serves as a predisposing risk-factor for increased PTSD symptomatology (Admon et al., 2013).

It should also be noted that vigilance to threat (e.g., angry faces) has long been associated with anxiety in general and PTSD in particular (Bar-Haim et al., 2007; Fani et al., 2012). However, in this study selective attention to threat was not directly associated with PTSS, perhaps due to the small sample size and low levels of symptoms. Nonetheless, our results do show that vigilance is related to the neural response of anger that predicts later PTSS, supporting the notion that selective attention to angry faces is related not only to anxiety, but also to anger (Van Honk et al., 2001). Indeed, it has been suggested that angry faces may induce fear or anger (Dimberg and Öhman, 1996), depending on how threat is appraised; when it is interpreted as dangerous it leads to fear, but when interpreted as provocative it may elicit anger (Beck, 1976). Interestingly, cortisol may play a role in mediating the tendency to interpret threatening stimuli as either provocative or dangerous; Studies indicate that cortisol administration may lead to increased vigilance towards angry faces and reduced vigilance towards fearful faces, while at the same time it may enhance the inhibition of irrelevant threatening stimuli (for review, see Putman and Roelofs, 2011). Importantly, fear, anger and under certain conditions also low levels of cortisol have been related to pathological anxiety including PTSD (Dunmore et al., 2001; Meewisse et al., 2007; Shin and Liberzon, 2009), calling for future studies to specifically address this intriguing and complex relationship.

### Limitations

Several limitations of this study should be taken into account. First, participants were healthy young male soldiers with sub-threshold PTSS levels. It is unclear to what extent the current findings generalize to other samples. It also remains to be demonstrated whether our results generalize to other types of stressors, including high intensity combat exposure. Unfortunately, baseline PCL symptoms and trauma history were not assessed, thus our results do not preclude effects of prior traumatic events. To resolve this issue, further prospective studies that assess symptoms prior to stress exposure are required. Another possible limitation is that in this study anger was elicited using only one specific film excerpt, which might have not induced the same anger response among all participants. Finally, the current findings indicate that the predictors accounted for 35% of the variance of PTSS. While this is promising, future research should consider additional factors such as trauma history and genetic markers that could improve prediction of outcome.

### Conclusion

Shedding light on the functional role of anger in PTSS, the current findings point to anger-related psychological and neural vulnerability factors that can be measured efficiently and at a low cost before stress exposure. Although replications of these results are required to draw firm conclusions, measurements of state-anger and amygdala activity evoked by an angering experience may eventually improve prevention and early intervention among individuals prone to stress and trauma exposure.

## Author Contributions

TL: conceptualization, data acquisition, formal analysis, writing—original draft, review and editing. GG: conceptualization, data acquisition, writing—review and editing. GR: conceptualization, formal analysis, writing—review and editing. AO-B: data analysis, writing—review and editing. YB-H: conceptualization, resources for data acquisition, writing—review and editing. EF: resources for data acquisition, writing—review and editing. TH: conceptualization, writing—original draft, review and editing, funding acquisition.

## Conflict of Interest Statement

The authors declare that the research was conducted in the absence of any commercial or financial relationships that could be construed as a potential conflict of interest.
